# Multi-Hop Question Generation Using Hierarchical Encoding-Decoding and Context Switch Mechanism

**DOI:** 10.3390/e23111449

**Published:** 2021-10-31

**Authors:** Tianbo Ji, Chenyang Lyu, Zhichao Cao, Peng Cheng

**Affiliations:** 1 School of Transportation and Civil Engineering, Nantong University, Nantong 226019, China; jitianbo@ntu.edu.cn (T.J.); caozhichao@bjtu.edu.cn (Z.C.); 2 Science Foundation Ireland Centre for Research Training in Machine Learning, School of Computing, Dublin City University, Dublin 9, Ireland; 3 Alibaba Group, Hangzhou 311121, China; chris.cp@alibaba-inc.com

**Keywords:** multi-hop question generation, hierarchical encoding-decoding, syntactic knowledge

## Abstract

Neural auto-regressive sequence-to-sequence models have been dominant in text generation tasks, especially the question generation task. However, neural generation models suffer from the global and local semantic semantic drift problems. Hence, we propose the hierarchical encoding–decoding mechanism that aims at encoding rich structure information of the input passages and reducing the variance in the decoding phase. In the encoder, we hierarchically encode the input passages according to its structure at four granularity-levels: [word, chunk, sentence, document]-level. Second, we progressively select the context vector from the document-level representations to the word-level representations at each decoding time step. At each time-step in the decoding phase, we progressively select the context vector from the document-level representations to word-level. We also propose the context switch mechanism that enables the decoder to use the context vector from the last step when generating the current word at each time-step.It provides a means of improving the stability of the text generation process during the decoding phase when generating a set of consecutive words. Additionally, we inject syntactic parsing knowledge to enrich the word representations. Experimental results show that our proposed model substantially improves the performance and outperforms previous baselines according to both automatic and human evaluation. Besides, we implement a deep and comprehensive analysis of generated questions based on their types.

## 1. Introduction

Question generation (QG) aims to generate appropriate questions for the given passages, it is an important task in natural language processing (NLP) research, QG has many applications for various NLP tasks. For example, QG can be used to augment a question answering (QA) dataset that is expensive to obtain, construct a synthetic QA dataset and facilitate a dialogue system by controlling conversation flow through generating questions. Besides, QG can be used for an educational purpose as it can improve and enhance children’s comprehension and retention by proposing questions based on textbook passages [1,2,3,4]. Especially, in the QG research community, multi-hop QG has recently been the focus of its potential applications in understanding complex human questions generated through the compositionality of questions, and the goal of multi-hop QG is to generate complex questions that require evidence across multiple passages to be answered [5].

QG has attracted researchers’ interests for many years. In the early years, rule/template-based methods were the mainstream models for the QG task. For example, a rule-based approach was proposed to transform a declarative sentence into its interrogative counterparts, and a statistical ranker was then invoked to select the most appropriate questions and discard those of low quality [6]. However, rule/template-based methods can only generate trivia questions by simply reordering clauses and manipulating words in the sentence, while it cannot handle complicated sentences. Since natural language is highly flexible, there are scenarios that rule/template-based approaches fail to process. Meanwhile, it is also difficult to accurately parse a sentence and obtain its constituents. To overcome such shortcomings, vector-based machine learning models have been introduced into QG tasks with the advent of the neural sequence-to-sequence (seq2seq) framework. This adds the advantages of modeling semantics of natural language in vector space and producing more fluent and human-like text [7]. After the deployment of neural networks in the QG tasks, various models were proposed and the quality of generated questions has been significantly improved, especially in terms of readability [8].

Despite the successful application in QG tasks, neural models still bear limitations and remain prone to generating irrelevant questions, particularly when producing complex questions according to multiple relevant passages. Usually denoted as *semantic drift* [9], such problems in QG can be categorized in two classes: global or local. In regard to the global semantic drift problem, a generated question might be grammatically correct, but its overall semantic meaning is irrelevant to the input passages and/or the answer. For example, given a set of passages about Isaac Newton together with the answer about the date when he was born, a neural QG may generate “When did Isaac Newton write the book Philosophiæ Naturalis Principia Mathematica?”, or even “Who wrote the book Philosophiæ Naturalis Principia Mathematica?”. Such generated questions are fluent and meaningful, but mismatch the answer and passages. Meanwhile, local semantic drift indicates that the semantic units (i.e., phrases or words) in the generated question are inconsistent with each other, resulting in the failure of forming a meaningful sentence. In this case, with the previous passages and the answer, the model-generated question might be “In the time of Dark Ages, who helped Isaac Newton invent the first electronic computer?”, where “Dark Ages”, “Isaac Newton” and “electronic computer” are neither compatible nor consistent.

To address the two aforementioned semantic drift problems, we propose two separate mechanisms: a hierarchical encoding–decoding mechanism and a context switch mechanism, which, respectively, seek to alleviate the global and local semantic drift problems. Inspired by the fact that the structural information on different granularity-levels has been proved to be helpful for encoding rich semantic information [10,11,12], we think a hierarchical structure is suitable for taking the advantage of the structural information. Following the typical seq2Seq framework, the hierarchical encoding–decoding mechanism also consist of an encoder and a decoder, where the former receives the input textual passages and encodes their structural information, and the latter receives the encoded information from the encoder and decodes the question in a coarse-to-fine fashion through the computation of attention weights. In detail, four various levels of granularity are involved during the encoding phase, including word-level, chunk-level, sentence-level and document-level, and the encoder will encode the textual passages based on their structure with these granularity-levels. Subsequently, the decoder will select the context vector in a coarse-to-fine fashion during the decoding phase: first selecting it on the document-level, then at the sentence-level and chunk-level, and finally on the word-level. Additionally, since the decoder generates words one by one in the decoding phase, we think the generated words in the same semantic unit (e.g., a phrase or an entity) should be more consistent and semantically related if they have similar context vectors. Thereby, we propose the context switch mechanism to provide similar context vectors when the QG model is expected to produce words in the same semantic unit. In the implementation of the context switch mechanism, an extra layer is included to output the probability at each decoding time-step, for the purpose of effectively using the context vector from the last step.

We then assess the performances of the proposed model and other baseline QG models by evaluating the results on the benchmark dataset HotpotQA [5]. Prevailing automatic evaluation metrics like such as BLEU [13], ROUGE [14] and METEOR [15] are employed, and we further conduct a human evaluation experiment since these automatic evaluation methods have been proven to have poor correlation with human [16]. The experimental results show the proposed model can improve the quality of generated questions according to both automatic and human evaluation.

## 2. Related Work and Background

### 2.1. Question Generation

The question generation task has been explored broadly in the early natural language processing work where it mainly focused on rule-based approaches using heuristics induced from linguistic knowledge (such as dependency parsing and constituency parsing) to manipulate constituents in the sentence to produce an interrogative sentence. For example, a rule-based framework that utilizes heuristics from syntactic knowledge is proposed to transform declarative sentences to corresponding questions as candidates [6]. A statistical ranking model is then employed to score the candidates, and those of low-quality will be discarded.

Thereafter, the neural seq2seq model becomes dominant in the QG task and has achieved high performances [17], because of the successful application of neural models in other text generation tasks (e.g., machine translation and question answering). An attention-based bidirectional long short-term memory (LSTM) model is employed to generate questions given a pair of passage and answer [8]. In order to produce questions that are relevant to the corresponding answers, Sun et al. [18] propose to incorporate the point-generator network [19] and the word embedding of an textual answer. Likewise, Ma et al. [20] propose a QG model that can strength the connections of passages, answers, and questions by matching the sentence-level semantics and predicting the answer position in the passage. Chen et al. [21] adopt a reinforcement learning approach to directly optimize the QG model according to discrete evaluation metrics for the purpose of bridging the gap among the training objective, the word-level optimization, the inference aim, and the generation of a sentence-level output. With the help of advanced linguistic parsers such as dependency parsing, semantic role labeling (SRL) and named entity recognition, Dhole and Manning [22] leverage templates to generate questions based on the parsing results, including a dependency tree and SRL frames. The proposed approach achieves state-of-the-art results on the SQuAD dataset [23], outperforming the previously proposed neural QG model, showing that QG can benefit from the incorporation of linguistic and syntactic knowledge.

There are some other works exploring different aspects of the QG task, such as incorporating question types [24], encoding wider context information [25] as well as the combination of QA and QG [26].

### 2.2. Multi-Hop Question Generation

The Multi-hop QG task has its own complexity since complex questions are generated from multiple interconnected input passages [5]. Gupta et al. [27] introduces reinforcement learning and multitask learning into multi-hop QG, which specifically treats answer position prediction and supporting facts prediction as two extra tasks in the seq2seq training process. Their experimental results show that the proposed approach achieves high performance compared to baseline models.

Pan et al. [28] use the semantic units parsed from semantic role labeling and dependency parsing to construct a semantic graph for documents in order to model the connections among the semantic units as well as the documents usually neglected in prior arts. Then, a recurrent neural network (RNN) encoder and a graph neural network (GNN) encoder are invoked to encode the documents. The representations generated by the RNN encoder show a document’s basic textual information while the representations from the GNN encoder are expected to contain semantic information enhanced by the graph structure induced by semantic role labeling and dependency parsing. Next, an attention-based decoder is used to generate the question word by word. The proposed semantic graph model outperforms previous work by a large margin.

Furthermore, Xie et al. [29] explore how question-specific rewards used in reinforcement learning relate to the quality of questions for multi-hop QG, three question-specific rewards—fluency, relevancy, and answerability—are proposed. From the perspective of human evaluation, the findings from the experimental results suggest that directly optimizing relevancy yields improvements on the question quality; however, optimizing the other two rewards—fluency and answerability—results in quality degradation of questions, especially for answerability.

### 2.3. Evaluation of Question Generation

Previous work on question generation mostly uses discrete metrics (BLEU, ROUGE, and METEOR) from general text generation tasks (such as machine translation and text summarization). Nevertheless, those metrics have been shown to have flaws in evaluating text generation tasks. The findings from Reiter [16] support the usage of BLEU in machine translation, but BLEU is not suitable for other text generation tasks especially when evaluating individual texts. Accordingly, evaluating question generation with such discrete metrics is inappropriate since there is only one reference question for each generated question resulting from the common practice of using a QA dataset for the QG task. Moreover, there may be multiple appropriate questions for the input passages and answer. Thus, metrics such as BLEU and ROUGE evaluating lexical similarity are not suitable for the question generation task.

Human evaluation is also widely applied in the assessment of QG models. A common practice is to randomly sample a few hundred generated questions and to ask human raters to evaluate them by different dimensions (i.e., adequacy and fluency) on a five-point scale [17]. The final result of human evaluation is reported as the ranking of models by their average rating scores.

### 2.4. Seq2seq Generation Model and Attention-Based Decoder

In this section, we introduce the basic structure of the seq2seq text generation model [7] and the attention-based decoder [30].

#### RNN-Based Seq2Seq Model

Given a source sequence $X=\{x_{1},x_{2},\ldots ,x_{n}\}$, a seq2seq text generation model is expected to generate a target sequence $Y=\{y_{1},y_{2},\ldots ,y_{m}\}$ where *x* and *y* are the tokens in sequences *X* and *Y*, respectively. A seq2seq model usually coheres with the encoder-decoder structure, where the encoder firstly receives the source sequence *X* as the input and produces representations of *X*, and the decoder can then generate the target sequence *Y* token by token using the previously produced representations of *X*.

A typical implementation of a RNN-based seq2seq model uses a RNN encoder and a RNN decoder to constitute its encoder–decoder structure. With the source sequence *X* that has *n* tokens, we feed its tokens one by one into the RNN encoder:

(1) $$h_{t}=g\left(h_{t-1},x_{t}\right)$$

where $h_{t}$ is the encoded representation for the hidden state at a time-step *t* and $x_{t}$ is the *t*-th token in the sequence *X*. Following the encoding phase, the decoder takes the last result provided by the encoder for the hidden state $h_{n}$ as the first decoder result for hidden states and generates each decoded hidden state one-by-one:

(2) $$s_{t}=f\left(s_{t-1},y_{t-1}\right)$$

where $s_{t}$ is the decoder result for hidden states at a time-step *t*, specifically $s_{0}=h_{n}$, $y_{t-1}$ is the t−1-th target token. To generate the target token at the *t* time-step, we use the decoder result for hidden state $s_{t}$ to obtain a probability distribution over the vocabulary. Thereafter, we select the one with the highest probability:

(3) $$y_{t}^{\prime }=softmax\left(s_{t},y_{t-1}\right)$$

It is noteworthy that in the training process, it is common to adopt the teacher-forcing mechanism [31] in which we directly input the ground-truth target token $y_{t-1}$ at the time-step *t* during the decoding phase instead of the last predicted token in order to stabilize the training process. In the inference process, we always input the predicted target token $y_{t-1}^{\prime }$ since the ground-truth target token $y_{t-1}$ is not available.

### 2.5. Attention-Based Decoder

In the vanilla RNN encoder–decoder structure, the encoder and decoder are independent as the only connection between them is that the latter uses the last hidden states from the former to initialize its own hidden states. The information of the source sequence unfortunately lack full utilization. Hence, Bahdanau et al. [30] propose an attention mechanism to enable the decoder to select the part of the source sequence on which to focus when generating target token. Concretely, an extra term *c* called context vector is added into Equation (2):

(4) $$s_{t}=f\left(s_{t-1},y_{t-1},c_{t}\right)$$

where $c_{t}$ is the context vector at the time-step *t*, which is computed using the combination of all encoder resulting hidden states:

(5) $$c_{t}=\sum_{i=1}^{n}e_{i,t}h_{i}$$

where $e_{i,t}$ is the normalized coefficient for decoder resulting hidden state $s_{t}$ and encoder resulting hidden states $h_{i}$. The equation for computing $e_{i,t}$ is:

(6) $$e_{i,t}=\frac{\phi (s_{t},h_{i})}{\sum_{j=1}^{n}\phi (s_{t},h_{j})}$$

where ϕ is the scoring function measuring the connection between $s_{t}$ and $h_{i}$.

## 3. Model Architecture

Our model is a bidirectional gated recurrent unit (GRU) [32,33] based RNN consisting of an encoder and a decoder. Given a set of documents $D=\{w_{1},w_{2},\ldots ,w_{v}\}$ and the answer $Ans=\{a_{1},a_{2},\ldots ,a_{u}\}$, our model receives the concatenation of *D* and Ans as the input, where *w* is a word in *D*, *a* is a word in Ans, and the input contains *n* words (n=v+u). Besides, we record the hierarchical information of the input using a document-sentence-chunk-word structure. In detail, *D* can be described as a combination of documents $D=\{doc_{1},doc_{2},\ldots \}$, where each document doc is $doc=\{sent_{1},sent_{2},\ldots \}$, each sentence sent is $sent=\{chunk_{1},chunk_{2},\ldots \}$ and each chunk chunk is $chunk=\{word_{1},word_{2},\ldots \}$.

**Figure 1:**
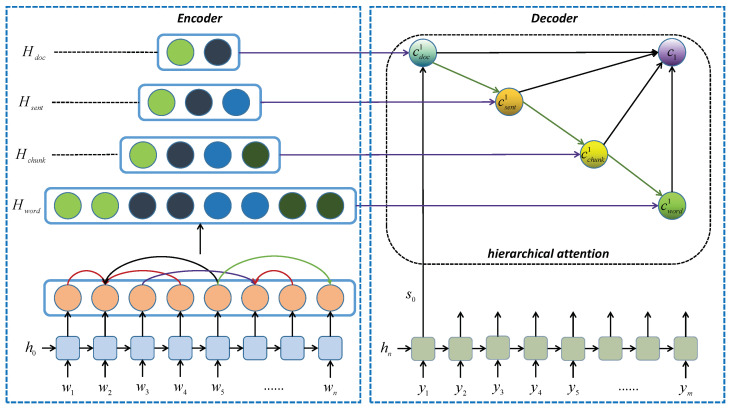
The structure of the proposed seq2seq model, including the encoder (**left**) and the decoder (**right**).

Figure 1 provides the overall architecture our proposed model and describes the generation process at the time-step t=1. The encoder firstly encodes the input documents to obtain its sequential representation $H_{seq}$ and injects dependency parsing into $H_{seq}$ to get the dependency representation $H_{dep}$, we therewith fuse $H_{seq}$ and $H_{dep}$ to form the word-level representation $H_{word}$. Afterwards, we can successively get the chunk-level, sentence-level and document-level representation ($H_{chunk}$, $H_{sent}$ and $H_{doc}$) according to the word-level representation $H_{word}$, and the details will be introduced in Section 3.1. Hence, we have representations of documents on four granularity-levels: $H_{word}\in R^{n\times h},H_{chunk}\in R^{num\_c\times h},H_{sent}\in R^{num\_s\times h},H_{doc}\in R^{num\_d\times h}$, where *h* means the length of each word representation, *n*, *num_c*, *num_s* and *num_d* represent the number of words, chunks, sentences and documents, respectively.

In the attention mechanism of decoding phase, we select the context vector in a coarse-to-fine way at each time-step. Specifically, we select the document-level context $c_{t}^{doc}$ at first, and it can be used to guide the selection of sentence-level context $c_{t}^{sent}$. Then, both $c_{t}^{doc}$ and $c_{t}^{sent}$ can help to select the chunk-level context $c_{t}^{chunk}$. At the end, we incorporate these three context vectors to select the word-level context $c_{t}^{word}$. Finally, we fuse these four context vectors to obtain a context vector $c_{t}$ at the time-step *t*, and it will be used to generate a word $y_{t}$ belonging to the vocabulary.

### 3.1. Encoder

The encoder first uses a bidirectional GRU network to encode the concatenated input texts [D,Ans] to obtain its sequential representation which is denoted as $H_{seq}\in R^{n\times h}$, and we use the last hidden states of answer tokens as its representation. Next, we will inject the dependency parsing information into $H_{seq}$ to obtain the dependency representation $H_{dep}$. Each word in a parsing tree has an ancestor node, and some words may have a child node, which means each word in a parsing tree has at least one edge connecting to another word, and such edge information can be used to incorporate with the dependency parsing information. Similar to a graph neural network (GNN), we encode such information as follows:

(7) $$\begin{array}{cc} w_{i}^{{}^{\prime }} & =\sum_{i=1}^{k}M_{k}w_{i}^{k} \end{array}$$

(8) $$\begin{array}{cc} w_{i} & =g(w_{i},w_{i}^{{}^{\prime }}) \end{array}$$

where $w_{i}^{k}$ is the word representation of the *k*-th word in $w_{i}$’s neighborhood words, $M_{k}$ is the transformation matrix of corresponding edge type connecting $w_{i}$ and $w_{k}$. Then, the function *g* updates the current word representation of $w_{i}$ using $w_{i}^{{}^{\prime }}$. We repeat Equations (7) and (8) for *T* turns to enable better message passing through word representations, where *T* is a hyper-parameter.

After the injection, we can generate a set of new word representations called the dependency representation $H_{dep}\in R^{n\times h}$. We then fuse $H_{seq}$ and $H_{dep}$ together to form a new sequential representation $H_{word}$ by concatenation. Using the word-level representation $H_{word}$, we can obtain other structural representations of the input [D,Ans] according to its alignment matrices:

(9) $$\begin{array}{cc} H_{chunk} & =\sigma (A_{chunk}^{T}H_{word}) \end{array}$$

(10) $$\begin{array}{cc} H_{sent} & =\sigma (A_{sent}^{T}H_{chunk}) \end{array}$$

(11) $$\begin{array}{cc} H_{doc} & =\sigma (A_{doc}^{T}H_{sent}) \end{array}$$

where $A_{chunk}\in R^{n\times h},A_{sent}\in R^{num\_c\times h},A_{doc}\in R^{num\_s\times h}$ are the chunk-level, sentence-level and document-level alignment matrices, respectively. Each entry in an alignment matrix *A* is either 1 or 0, which represents whether a column in the representation *H* should be included in the current chunk/sentence/document or not. For example, an entry $A_{chunk}^{ij}\in A_{chunk}$ can indicate whether the *j*-th word in $H_{word}$ should be included in the *i*-th chunk ($A_{chunk}^{ij}=1$) or not ($A_{chunk}^{ij}=0$), where *i* and *j* are the row and the column the entry located in.

### 3.2. Decoder

Following a typical auto-regressive setup, our model can compute the context vector $c_{t}$ through an attention function with the current hidden states $h_{t}$ to generate a word at a time. Specifically, we combine the last hidden states resulting from the encoder to form the initial hidden states $s_{0}$. Different from a vanilla attention-based auto-regressive decoder described in Section 2.5, our decoder is equipped with a hierarchical attention function in which the context vectors are generated in accordance with the coarse-to-fine fashion (from document-level to word-level). Concretely, at a time-step *t* during the decoding phase, the context vectors in various levels of granularity are generated as follows:

(12) $$\begin{array}{cc} c_{t}^{doc} & =attention(s_{t-1},H_{doc}) \end{array}$$

(13) $$\begin{array}{cc} c_{t}^{sent} & =attention(\left[s_{t-1},c_{doc}\right],H_{sent}) \end{array}$$

(14) $$\begin{array}{cc} c_{t}^{chunk} & =attention(\left[s_{t-1},c_{doc},c_{sent}\right],H_{chunk}) \end{array}$$

(15) $$\begin{array}{cc} c_{t}^{word} & =attention(\left[s_{t-1},c_{doc},c_{sent},c_{word}\right],H_{word}) \end{array}$$

where the $s_{t-1}$ is the hidden states resulting from the decoder at the time-step t−1, $\left[s_{t-1},c_{doc}\right]$ is the concatenation operation to combine $s_{t-1}$ and $c_{doc}$ together, and the attention function follows Equations (4)–(6). Then, we use the fuse function to obtain the final context vector $c_{t}$ at the time-step *t* using these four computed context vectors $c_{t}^{doc}$, $c_{t}^{sent}$, $c_{t}^{chunk}$ and $c_{t}^{word}$:

(16) $$c_{t}=fuse\left(c_{t}^{doc},c_{t}^{sent},c_{t}^{chunk},c_{t}^{word}\right)$$

Finally, we generate the decoder hidden states $s_{t}$ based on the embedding of the last word $y_{t-1}$, the context vector $c_{t}$ and the previous hidden states $s_{t-1}$, then the word $y_{t}$ at the time-step *t* is generated based on $s_{t}$ and the previous word $y_{t-1}$:

(17) $$\begin{array}{cc} s_{t} & =f(y_{t-1},c_{t},s_{t-1}) \end{array}$$

(18) $$\begin{array}{cc} y_{t} & =softmax(y_{t-1},s_{t}) \end{array}$$

### 3.3. The Context Switch Mechanism

**Figure 2:**
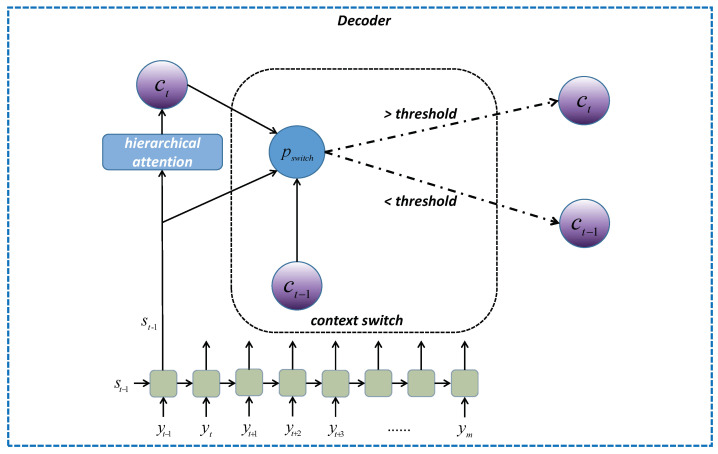
The structure of the context switch mechanism employed in our model.

Moreover, in order to increase the stability of the decoding process, we add the context switch mechanism that enables sharing similar contexts through a set of words when they are generated consecutively. Figure 2 represents the structure of the context switch mechanism as well as the working process at the time-step *t*.

For the implementation of this mechanism, an extra linear layer is included to produce a probability $p_{switch}$ as the indication of the selection between using the previous context vector $c_{t-1}$ and keeping the current one $c_{t}$. The probability $p_{switch}$ is computed by the following equation:

(19) $$p_{switch}=\psi \left(s_{t},c_{t},c_{t-1}\right)$$

where the function ψ uses $c_{t-1}$, $c_{t}$ and the current hidden states $s_{t}$ from the decoder to produce the probability. For $p_{switch}\geq \alpha$, the $c_{t}$ will be replaced by $c_{t-1}$ in Equation (17); otherwise, $c_{t}$ remains, where α is the value of threshold we predefined. In practice, α is set to 0.5, as we think activating $p_{switch}$ or not should be equiprobable.

### 3.4. Training Objective

Generally speaking, the training objective of a seq2seq model is to maximize the probability of the target sequence $Y=\{y_{1},y_{2},\ldots ,y_{m}\}$ when given the source sequence $X=\{w_{1},w_{2},\ldots ,w_{n}\}$, as described in Equation (20):

(20) $$\begin{array}{cc} P(Y|X) & =P(y_{1},y_{2},\ldots ,y_{m}|w_{1},w_{2},\ldots ,w_{n})\\ \; & =\prod_{t=1}^{m}P\left(y_{t}|H,y_{1},y_{2},\ldots ,y_{t-1}\right) \end{array}$$

where *H* is the representation of the source sequence *X* and $y_{t}$ is conditioned on the generated tokens before time-step *t*. To maximize the probability P(Y|X), we train our model using negative log likelihood loss (NLLLoss) for the generation objective:

(21) $$L\left(\theta \right)=-\frac{1}{m}\sum_{t=0}^{m-1}logp\left(y_{t}|H,y_{<t};\theta \right)$$

where θ represents the parameters of our model. We employ *Adam* [34] optimizer to optimize the parameters with c.

## 4. Experiments

### 4.1. Data Reparation

In this paper, we conduct experiments on HotpotQA [5], which is a multi-hop question and answering dataset (https://hotpotqa.github.io (accessed on 15 August 2021)). The terminology *multi-hop* means it requires a QA model to reason over multiple passages and grab corresponding information to answer the questions in the HotpotQA dataset. For the usage of the HotpotQA dataset in the QG task, the QG model will take an answer as well as its related passages to generate a question. The original HotpotQA dataset consists of 〈passage,answer,question〉 tuples, and is split into two sets for training (90564 samples) and testing (7405 samples), respectively. We extract the annotated supporting facts sentences in passages rather than the whole passages as the input to our model. To obtain the dependency trees and constituency trees (chunk-level information) of the documents in train and test set, we employ the AllenNLP [35] dependency parser (https://demo.allennlp.org/dependency-parsing (accessed on 10 September 2021)) and constituency parser (https://demo.allennlp.org/constituency-parsing) (accessed on 10 September 2021).

### 4.2. Training and Inference Setup

The detailed hyper-parameters for training our model are selected as follows: (1) the learning rate is 7.5 × 10^−4^; (2) the weight decay rate is 0; (3) the batch size is 32; (4) the dropout rate is 0.4; and (5) the maximum gradient norm is 5. We employ the global vectors for word representation (GloVe) [36], where the dimension of word embedding is 300, both the encoder hidden size and decoder hidden size are set to 768. Furthermore, the number of turns *T* for injecting the dependency information are set to 3. During the inference phase, we input the test set into the trained hierarchical encoding–decoding model while the size of beam search is set to 5.

### 4.3. Evaluation

#### 4.3.1. Evaluated Models

To analyze the performance of our proposed model and the quality of the generated questions, we will compare the performance with baseline models. Six models are involved for the comparisons, as described as follows:

- **Our model-1**: Our proposed hierarchical encoding-decoding QG model;
- **Our model-2**: The proposed QG model integrated with a larger dictionary that mitigates all unknown tokens;
- **Semantic-Graph**: A framework that contains semantic graphs and an encoder using an attention-based gated graph neural network [28];
- Semantic-Graph*: Semantic-Graph with the context switch mechanism;
- **RNN**: A vanilla RNN-based seq2seq model;
- **GPT-2**: A large transformer-based language model [37].

#### 4.3.2. Automatic Evaluation

We use the following prevalent evaluation metrics to automatically assess the performances of question generation models:

- **BLEU-N**: A method that measures the precision based on the n-gram overlap between generated questions and references [13]. We compute BLEU-[1,2,3,4] in this experiment.
- **ROUGE-L**: ROUGE-L is a method that measures precision and recall on the longest common subsequence (LCS) overlap between system outputs and references [38].
- **METEOR**: METEOR uses a set of stages (e.g., word stemming, synonyms, etc.) to generate the mappings of unigrams between system outputs and references, and compute the weighted harmonic mean of precision and recall based on the mappings [15]. Recall has a higher weight than precision.

Table 1 represents the metric scores of these QG models. Compared to the baselines, we find that our proposed QG model with a larger dictionary (Our model-2) outperforms other models according to ROUGE-L, the proposed model with or without the dictionary can outperform the current state-of-the-art model *Semantic-graph* on ROUGE-L. In particular, our model outperforms the large pre-trained language model GPT-2 on both ROUGE-L and METEOR. We also find Semantic-Graph* has the highest METEOR and BLEU-1 score, which clearly proves the effectiveness of our proposed context switch mechanism that has been applied in the *Semantic Graph* model. However, the GPT-2 models have the best performance on BLEU-[2,3,4].

#### 4.3.3. Human Evaluation

Since the popular automatic metrics appear to not agree with each other, we additionally examine the human evaluation as a means of further investigating the performances of these QG models. We conduct the crowd-sourcing experiment on Amazon Mechanical Turk (https://www.mturk.com/ accessed on 11 October 2021), and we ask human workers to evaluate the performances of seven models, including the previous six QG models and an extra model, Gold, for which the outputs consist of the reference questions.

For the judgment of a single generated question, a PAQ tuple 〈p,a,q〉 (p= paragraph, a= answer, q= question) will be shown to a human rater, and the rater is asked to judge the quality of the question according to four aspects: fluency, relevance, answerability, and complexity. In our experiment, each human rater is assigned with 15 PAQ tuples, and these questions are randomly selected from the outputs of the seven systems. We employ a 7-point rating scale (0–6) for every aspect that can be construed as: very bad, bad, fairly bad, indifferent, fairly good, good, and very good. We have involved 188 human raters comprising a total of 2820 evaluated outputs, and on average, a model is expected to have about 400 evaluated questions, which we believe is an appropriate sample size for human evaluation.

The result of human evaluation is reported in Table 2, where *N* is the number of rated system-generated questions, the overall score is computed by the arithmetic mean of fluency, relevance, answerability, and complexity scores. Systems are ranked by the overall score. We can observe that the model Gold has the best overall performance as expected, while our model-2 can outperform all other models. Furthermore, the performances of our model 2 with respect to the four separate aspects—of which fluency can even reach the level of *very good* (5–6)—are better than the other five QG models, when other models are only deemed to be *good* (4–5).

#### 4.3.4. Questions of Different Types

We split the questions into seven types: *What, Which, Who, How, Where, When* and *Other (questions without specific interrogative words)*, and analyze how the QG models prefer to to generate questions of a certain type from these types. Table 3 shows the percentage of question types in system-generated outputs, where reference represents the original data set. We find that the types of questions generated by these QG models is mostly similar to the reference questions. Furthermore, our models and the RNN model are prone to generating *What* questions, while GPT-2 generates 10% less *What* questions than the dataset. Among these models, RNN is the only model that generates no *Where* question.

To take a closer look at the quality of question types, we investigate the overall scores of human evaluation on different types of generated questions. According to the results of Table 1 and Table 2, ROUGE-L is the metric that correlates best with human scores. Thus, the ROUGE-L scores of different question types are also computed. Table 4 and Table 5 show the ROUGE-L and human evaluation scores of systems on our test data divided by different question types.

With respect to ROUGE-L scores shown in Table 4, although GPT-2 has the best quality on *Other*, Semantic-Graph and Semantic-Graph* achieve the best quality on *Where* and *When*. However, our proposed model with the dictionary (model-2) is able to generate *What*, *Which*, *Who*, and *How* questions with the best quality among all models. According to human evaluation, our model-2 outperforms the other question generation models on *What*, *Who* and *Other* questions, especially on *Other* questions. It warrants noting that the vanilla RNN model achieves the highest performance on *Which, How* and *When* questions. For *Where* questions, our model-2 and RNN get no human score because no question of this type is evaluated.

## 5. Discussion and Future Work

Although our model achieves a superior performance over other baseline models, there is still room for improvement, as Table 5 indicates that our model unfortunately performs worse than some other models on *Which, How* and *When* questions. Hence, how to incorporate more information in contextual encoding and decoding will be the future direction to be explored.

Besides, current QG models mainly focus on generating questions based on textual input, but the usage of input in other formats (e.g., images, audios and videos) receives less attention. For example, visual QG is a QG problem that takes images as the input, and its applications are also useful for the educational purpose, including child education and interactive lectures [39]. Our further attempt will involve combining our proposed QG model with image understanding approaches [40], and we believe it can be used to generate questions on visual arts for the purpose of helping children with their ability to appreciate art.

## 6. Conclusions

In this paper, we propose a novel question generation model incorporating the hierarchical encoding–decoding structure in order to inject the structural information of input documents, and a context switch mechanism for the purpose of stabilizing the decoding and making the generation process more consistent. The automatic metric results in Table 1 show our model achieves the best performance against baseline models on ROUGE-L in automatic metrics evaluation, although our model does not outperform baseline models on the other baseline models. Nonetheless, the results in Table 1 prove that our proposed context switch mechanism improves the model’s performance on automatic metrics. Furthermore, the human evaluation results also show our model outperforms all baseline models on four criteria we used. The experimental results of both automatic evaluation and human evaluation support the effectiveness of our proposed approach on the multi-hop QG task. In addition, we also conduct extensive studies analyzing the model’s performance on different question types according to both automatic evaluation metrics and human evaluation scores. Future work will include incorporating our method into pre-trained language models.

The data presented in this study are available in the Supplementary Materials.

## Figures and Tables

**Table 1:** Results of different QG models on the HotpotQA testset, the evaluation metrics are ROUGE-L, METEOR and BLEU-[1,2,3,4]. A score in bold indicates the model performs best according to that metric.

Model	ROUGE-L	METEOR	BLEU-1	BLEU-2	BLEU-3	BLEU-4
Our model-2	**27.34**	18.25	26.48	13.87	8.46	5.54
Our model-1	26.92	17.66	26.60	13.69	8.34	5.47
RNN	26.43	16.56	25.21	13.06	8.03	5.37
Semantic-Graph*	26.06	**20.71**	**27.20**	14.41	9.02	6.05
GPT-2	26.82	16.62	27.01	**17.72**	**12.49**	**9.14**
Semantic-Graph	25.74	20.32	26.55	13.94	8.57	5.56

**Table 2:** Results of the human evaluation experiment, where the overall score is the mean of fluency, relevance, answerability, and complexity, and *N* is the number of collected ratings. A score in bold means a model besides Gold performs best according to that evaluation aspect.

Model	*N*	Overall	Fluency	Relevance	Answerability	Complexity
Gold	408	5.05	5.16	5.00	5.09	4.97
Our model-2	395	**4.96**	**5.01**	**4.94**	**4.93**	**4.94**
Our model-1	417	4.86	4.91	4.87	4.80	4.87
RNN	382	4.83	4.79	4.89	4.80	4.85
Semantic-Graph*	394	4.74	4.63	4.80	4.79	4.76
GPT-2	418	4.69	4.77	4.72	4.52	4.77
Semantic-Graph	406	4.62	4.64	4.68	4.57	4.58

**Table 3:** The proportion (%) of question types in the outputs from different models. Question types are ordered by the results of reference.

Model	What	Which	Who	Other	How	Where	When
Reference	40.8	23.0	15.9	10.1	4.1	4.0	2.2
Our model-2	55.2	16.6	16.1	8.4	0.6	0.3	3.0
Our model-1	49.6	23.5	13.8	9.4	1.0	0.9	2.1
RNN	49.7	29.7	11.1	7.8	0.4	0.0	1.5
Semantic-Graph*	37.7	21.3	17.0	14.7	4.1	4.0	1.4
GPT-2	30.2	26.1	11.1	26.4	2.2	2.4	1.8
Semantic-Graph	36.2	20.2	15.4	18.7	3.2	2.9	3.4

**Table 4:** ROUGE-L scores on question of different types. A score in bold means a model has the highest ROUGE-L score on that question type.

Model	What	Which	Who	Other	How	Where	When
Our model-2	**26.57**	**29.86**	**26.73**	28.32	**30.23**	25.49	27.76
Our model-1	26.50	27.15	25.96	29.29	29.16	25.86	29.38
RNN	25.92	26.98	24.90	30.06	20.19	-	26.34
Semantic-Graph*	26.05	25.80	25.40	25.67	29.93	26.25	**31.16**
GPT-2	24.15	27.03	23.50	**30.60**	27.26	23.92	29.16
Semantic-Graph	26.11	26.24	24.74	24.76	28.79	**27.96**	24.07

**Table 5:** The overall human score on questions of different types. A score in bold means a model has the highest human evaluation score on that question type.

Model	What	Which	Who	Other	How	Where	When
Our model-2	**4.97**	4.80	**4.94**	**5.27**	5.63	-	4.82
Our model-1	4.92	4.76	4.85	4.60	5.67	5.00	5.15
RNN	4.81	**4.95**	4.68	4.67	**6.00**	-	**5.50**
Semantic-Graph*	4.76	4.62	4.86	4.88	4.47	4.84	3.94
GPT-2	4.95	4.61	4.36	4.59	5.61	**5.28**	4.04
Semantic-Graph	4.63	4.79	4.59	4.50	4.42	4.72	4.17

## Supplementary Materials

The following are available at https://www.mdpi.com/article/10.3390/e23111449/s1.
